# A Multimodal Deep Learning Approach to Intraoperative Nociception Monitoring: Integrating Electroencephalogram, Photoplethysmography, and Electrocardiogram

**DOI:** 10.3390/s25041150

**Published:** 2025-02-13

**Authors:** Omar M. T. Abdel Deen, Shou-Zen Fan, Jiann-Shing Shieh

**Affiliations:** 1 Department of Mechanical Engineering, Yuan Ze University, Taoyuan 320, Taiwan; omartalab40@gmail.com; 2 Department of Anesthesiology, En Chu Kong Hospital, New Taipei City 237, Taiwan; 3 College of Medicine, National Taiwan University, Taipei 100, Taiwan

**Keywords:** nociception prediction, multimodal monitoring, machine learning, EEG, PPG, ECG, anesthesia, LSTM, MLP

## Abstract

Monitoring nociception under general anesthesia remains challenging due to the complexity of pain pathways and the limitations of single-parameter methods. In this study, we introduce a multimodal approach that integrates electroencephalogram (EEG), photoplethysmography (PPG), and electrocardiogram (ECG) signals to predict nociception. We collected data from patients undergoing general anesthesia at two hospitals and developed and compared two deep learning models: a Multilayer Perceptron (MLP) and a Long Short-Term Memory (LSTM) network. Both models were trained on expert anesthesiologists’ assessments of nociception. We evaluated normalization strategies for offline and online usage and found that Min–Max normalization was most effective for our dataset. Our results demonstrate that the MLP model accurately captured nociceptive changes in response to painful surgical stimuli, whereas the LSTM model provided smoother predictions but with lower sensitivity to rapid changes. These findings underscore the potential of multimodal, deep learning-based solutions to improve real-time nociception monitoring in diverse clinical settings.

## 1. Introduction

Pain and nociception are often confused, but they are distinct. Pain is a conscious, subjective experience [1]. Under general anesthesia, however, “pain” is more accurately referred to as “nociception”, which reflects the physiological response to noxious stimuli in the absence of conscious perception. In the last two decades, significant progress has been made in developing methods to monitor nociception during anesthesia, particularly through algorithms that assess the nociception–antinociception (NAN) balance based on hemodynamic (e.g., ECG, PPG) and neural (e.g., EEG) signals.

An increase in the sympathetic tone might be perceived as a reaction to painful stimuli [2], while conversely, an increase in the parasympathetic tone might be related to analgesia. The Surgical Pleth Index (SPI) is derived from PPG signals and used to monitor NAN; it uses a combination of normalized heartbeat intervals (HBI_Norm_) and plethysmography amplitude (PPGA_Norm_) [3]. SPI is effective for nociception monitoring; however, since it is based on autonomic nervous system (ANS) activity, it might be confounded by other factors such as arrhythmia or antihypertensive drugs [4].

The analgesia nociception index (ANI), another measure used to monitor NAN, is derived from high-frequency heartbeat intervals [5,6]. The ANI is based on parasympathetic reactions and reflects noxious stimulations; however, it is also prone to arrhythmia and affected by parasympathetic reactions not related to pain. Moreover, the ANI was found to have a large inter-individual variability [4].

In general anesthesia, EEG signals are used to predict the consciousness level; however, some studies show that EEG-derived indexes (e.g., BIS, SE, and RE) increased significantly in painful stimulus events [7,8,9], and thus, it might be helpful to provide information about nociception. qNOX is another index that is derived from EEG signals [10]. It was developed using an Adaptive Neuro Fuzzy Inference System (ANFIS) by fitting different EEG frequency bands into a reference scale to predict noxious stimuli responses. Other methods have used single or a combination of parameters to monitor nociception [11,12].

Pain/nociception is a subjective experience [13]. Even with the absence of a clinical response, brain and spinal activities persist [14]. Pain perception starts when nociceptors sense painful stimuli. Nociceptors send pain signals through the spinal cord to the brainstem, where the first stage of processing takes place. These signals then move upward to the Thalamus, which serves as a central relay station for sensory information. Further processing and relay occur in higher brain regions, including the frontal cortex, specifically the somatosensory cortex and anterior cingulate cortex [15].

EEG bands were reported to react to several kinds of painful stimuli. It was found that EEG patterns are altered by noxious procedures in children, with local anesthesia mitigating this response [16]. Additionally, under sevoflurane anesthesia, the neuromuscular block affects BIS and EEG responses to noxious electrical stimulation, suggesting genuine arousal differences [17]. Clinical observations during tracheal intubation highlight alpha power changes as a sensitive indicator of cerebral activity modulation [18]. Moreover, noxious stimulation affects cortical electrical activity levels, as shown by EEG parameters [19].

The model selection process is challenging. Different models and methods were used in previous studies. For instance, SPI uses a linear model [3], while qNOX was developed using an ANFIS model [10], and the nociception level index was developed using random forest and linear regression models [11]. Long Short-Term Memory Neural Network (LSTM) models [20] are known for their ability to retain important information over a long period; therefore, they have the advantage of using previous knowledge to make predictions efficiently. On the other hand, Multilayer Perceptron (MLP) models do not need previous knowledge [21], and yet they can predict nonlinear relationships and find hidden patterns within the data [22].

While previous methods for nociception monitoring have demonstrated utility, the limitations of single-source approaches, particularly those reliant solely on the autonomic nervous system (ANS), prompt consideration of multimodal methods. ANS-based models, although informative, may be susceptible to non-nociceptive factors, potentially compromising the accuracy of nociception assessments. Moreover, the use of EEG data in isolation may not provide a comprehensive understanding of nociceptive states. In contrast, multimodal monitoring could offer the advantage of encompassing a broad spectrum of biomedical and clinical information. By integrating multiple physiological signals, such as EEG, PPG, and ECG, multimodal models have the capacity to capture diverse aspects of pain processing. Based on these considerations, we aim to address this challenge by implementing a multimodal approach utilizing EEG, PPG, and ECG signals to predict nociception during general anesthesia by integrating these three signals into one model. This study uses expert anesthesiologists’ nociception assessments (NOAs) based on clinical signs noted carefully during surgeries.

## 2. Materials and Methods

### 2.1. Data Collection

This work received approval from the institutional review board of the National Taiwan University Hospital (NTUH), and informed consent was obtained from all participants involved in this study. ECG, EEG, and PPG signals were collected using a Philips IntelliVue MP60 physiological signal monitor (Koninklijke Philips N.V, Amsterdam, The Netherlands), and the data were stored on a personal computer for further analysis. This study initially enrolled 142 patients from NTUH scheduled for general anesthesia surgery with inhalation anesthesia, during which propofol and fentanyl were administered. Another dataset from ECKH (*n* = 10) was included in the analysis when it became available, the signals were collected using CARESCAPE B650 monitor (GE Healthcare Finland Oy, Helsinki, Finland); the anesthesia method was also inhalation anesthesia. Due to missing signal data in the NTUH dataset, the final analysis included 90 patients from NTUH and 10 patients from ECKH. The patients’ demographic data are listed in Table 1.

Currently, no universally accepted gold standard for nociception exists, making assessment more challenging. The NOAs were scored based on each patient’s anesthesia record. The assessments were later digitized and stored in text files. After the surgeries were finished, each expert anesthesiologist (five in total) was appointed to assess the pain based on the information contained in the anesthesia record (e.g., HR, BP, anesthetic gas concentration, opioid dosages, etc.), and events, such as the eyelash reflex and laryngeal mask airway, were noted. As each anesthesiologist assessed pain individually, we synchronized the pain assessment data collected from multiple doctors and aligned them with the corresponding ECG, PPG, and EEG signals for analysis. Despite potential variations in assessment start times in a few cases, we matched data durations by comparing each patient’s assessment times and excluding offsets. The synchronization algorithm identified the latest start time (L) and the earliest end time (U) across all assessments, defining the analysis time interval [L, U]. Data points outside this interval were removed, ensuring only synchronized data were retained. Similar procedures were applied to align ECG, PPG, and EEG signals with synchronized pain score data. This process ensured consistency and the alignment of data across assessments.

**Figure 1:**
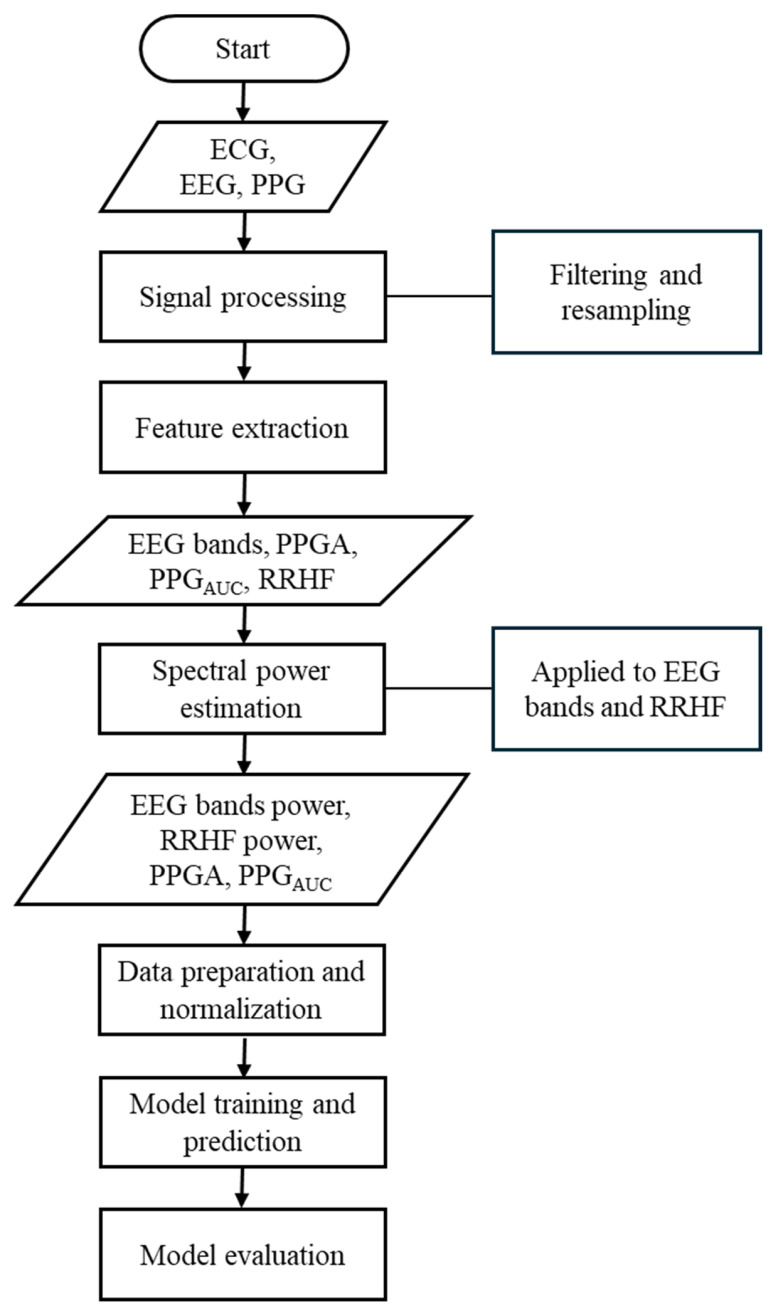
Flowchart of the methods used in this study. This flowchart briefly lists the methods used in this study.

Our study used different parameters extracted from each signal. The ECG signals were used to extract the high-frequency waveform of heartbeats (RRHF) and its spectral power (RRHF_PS_). The photoplethysmographic pulse wave amplitude (PPGA) and the area under the curve of PPG signals (PPG_AUC_) were also extracted. Additionally, EEG signals were used to calculate the spectral power for each frequency band (i.e., delta, theta, alpha, beta, and gamma). The proposed model in this study is described in the flowchart shown in Figure 1. All processing steps were conducted using MATLAB (version R2022b, MathWorks Inc., Natick, MA, USA) with the Signal Processing Toolbox.

### 2.2. Signal Processing and Feature Extraction

**Figure 2:**
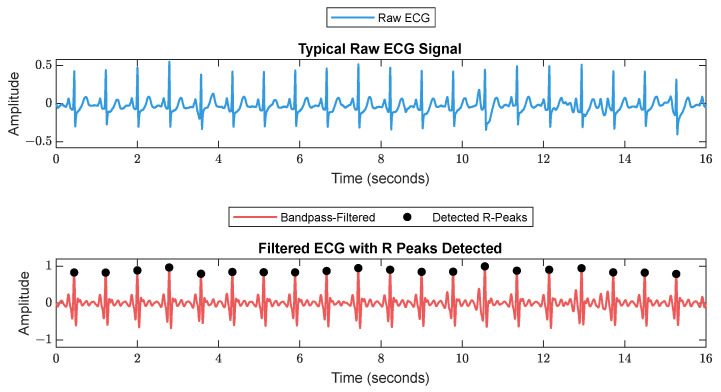
A 16 s ECG signal before (**upper plot**) and after (**lower plot**) filtering. The RR is calculated as the duration between each 2 successive R peaks.

**Figure 3:**
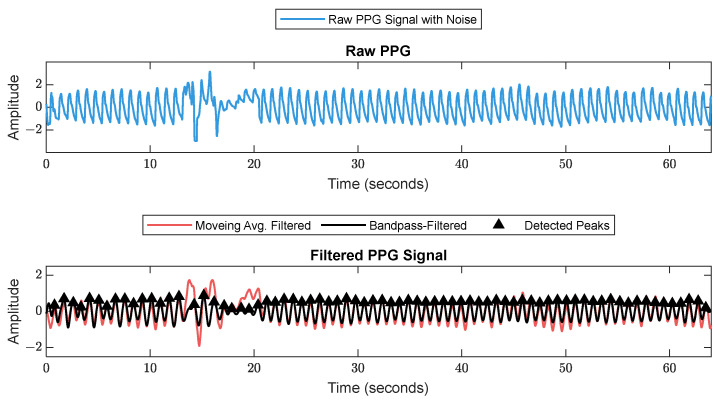
Raw PPG signal before (**upper plot**) and after (**lower plot**) filtering. The figure shows how the filter scheme enhanced some of the corrupted waveforms.

First, RR series were extracted from ECG (512 Hz) using Pan–Tompkin’s algorithm [23]. Then, we applied wavelet decomposition to the raw data and reconstructed only the RRHF in the range of 0.15–0.5 Hz, which is related to parasympathetic activity. Moreover, we calculated the power spectrum for the RRHF series over 64 s segments with a Kaiser window of 16 s. The PPG signals with a 128 Hz sampling frequency were filtered as needed. We used a Chebyshev II fifth-order filter with a cutoff of 0.5–8 Hz. For peak detection, we used the automatic multiscale-based peak detection algorithm [24,25]. The long-term trend of PPGA was more important than the instant value changes; therefore, we applied a moving average filter of a 16 s window on the raw data. The PPG_AUC_ was calculated for each 64 s window. Figure 2 shows a bandpass-filtered ECG window with its corresponding R peaks detected, and Figure 3 shows a PPG 64 s window before and after filtering.

**Figure 4:**
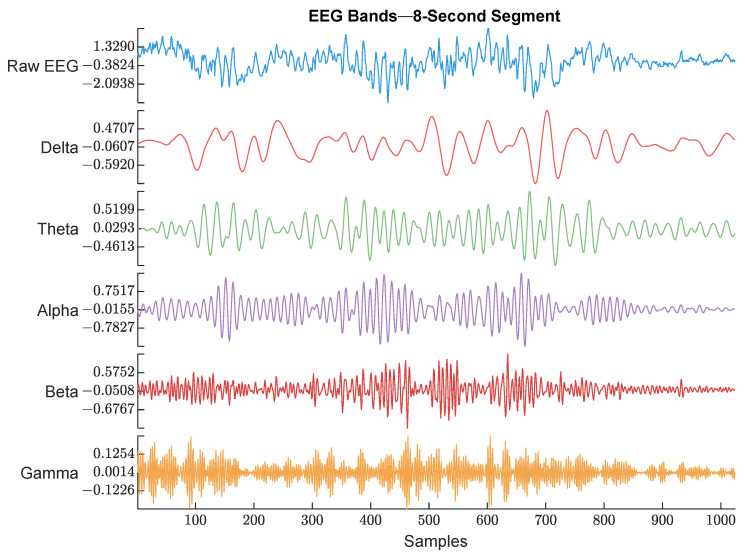
Raw EEG signal and its extracted bands. The frequency range used for each band is described below. The figure shows an example of each EEG band with different color. The waveforms are labeled with their corresponding band from top to bottom (Raw EEG, Delta band, Theta band, Alpha band, Beta band, and Gamma band).

EEG (128 Hz) was bandpass filtered between 0.5 and 48 Hz to keep the signal of interest that covered all the EEG bands. Then, the power spectrum was calculated by thresholding over each band’s frequency limits. An example of the raw EEG and its 5 bands are shown in Figure 4.

**Figure 5:**
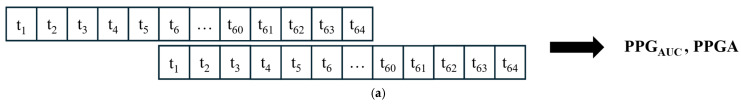

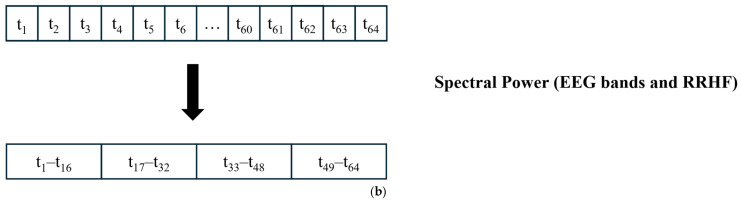
Data segmentation. (**a**) All signals were divided into 64 s windows. The windows overlapped with 59 s. After the first window is processed, the next window contains 59 s samples from the previous window and new 5 s samples. (**b**) For power calculations, the EEG and RRHF 64 s windows were divided into four segments of 16 s durations.

The EEG signal was first divided into 64 s segments with a 5 s time step; then, for each segment, the power was calculated using a Kaiser window of 16 s with no overlapping. Figure 5 shows the procedure for data windowing. The resulting power array was then averaged to obtain one value for each 64 s window. Although this is not standard, the used limits were as follows:

- Delta: 0.5–3.5.
- Theta: 4–7.5.
- Alpha: 8–12.
- Beta: 13–30.
- Gamma: 30.5–48.

### 2.3. Normalization

Initially, the histogram normalization described in [3] was used with a few adjustments. First, the standard deviation for the group dataset was not fixed; instead, it was the actual measure of the dataset. Second, the original distribution parameters were not combined; however, the transformation was calculated based on two distributions. The first transformation was based on the group dataset’s cumulative distribution function (cdf), and the second transformation was based on an accumulated individual dataset. At the beginning of the surgery, the weight for the group transformation was 100%. As more data became available, the weight for group data was reduced linearly until 10 min of the individual data was accumulated. The weight after that was fixed at 0.7 for individuals and 0.3 for groups. The transformations were calculated as:

(1) $$X_{norm}=w\cdot F_{x}\left(x\left(t\right)\right)+(1-w)\cdot F_{y}(x\left(t\right)),$$

where

- *X_norm_* is the normalized value of $x_{ij}$.
- $F_{x}(x(t))$ is the cumulative probability of x(t) in the individual dataset (from the beginning of the surgery until the current value).
- $F_{y}(x(t))$ is the cumulative probability of x(t) in the group dataset.
- w is the weight for the individual dataset, adjusted based on *t*, which is the window number. w starts at 0 and smoothly adjusts to 0.7 over the segments.

**Figure 6:**
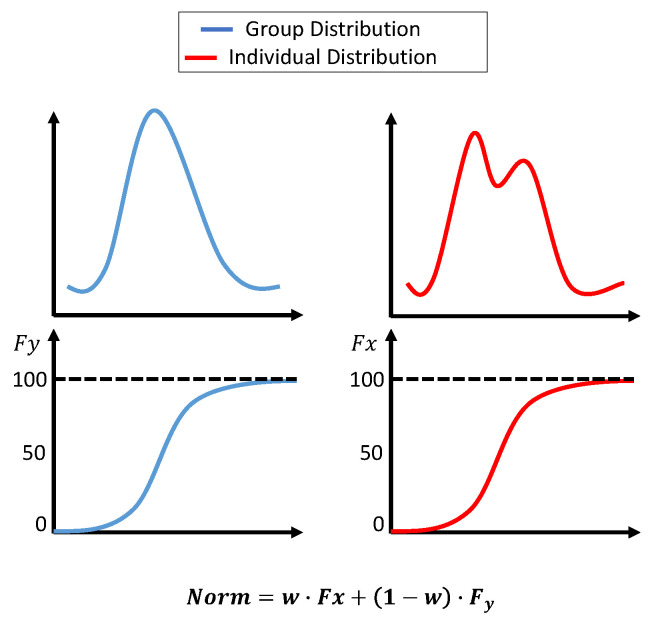
Histogram-based normalization. The **upper plots** show the dataset’s distribution, and the **lower plots** show the cdf. The equation in the figure shows the weights assigned to each transformation.

The histogram normalization is modeled in Figure 6.

When the data were normalized using histogram normalization, we noticed that the correlation between the normalized features and the pain assessment might be affected. Moreover, the variability in the original features was negatively affected in some cases; therefore, we decided to use Min–Max and z-score normalizations. The implementation was similar to histogram normalization, where the data were separated into group and individual datasets and the weights changed linearly based on the collected individual data from 100% to 30% for the group dataset. The z-score was previously utilized for online data normalization [11], and the equation in our study is as follows:

(2) $$X_{norm}=w_{1}\cdot \left(\frac{x\left(t\right)-mean\left(x_{accumulated}\right)}{std\left(x_{accumulated}\right)}\right)+w_{2}\cdot \left(\frac{x\left(t\right)-mean\left(x_{group}\right)}{std\left(x_{group}\right)}\right),$$

where

- *X_norm_* is the normalized value of x(t).
- $x_{accumulated}$ is the collected data from the beginning of the surgery to the current time.
- $x_{group}$ is the group dataset.

For Min–Max normalization, we followed a similar procedure as implemented in [3,11]; the used equation is:

(3) $$X_{norm}=w_{1}\cdot \left(\frac{x\left(t\right)-min\left(x_{accumulated}\right)}{max\left(x_{accumulated}\right)-min\left(x_{accumulated}\right)}\right)+w_{2}\cdot \;\left(\frac{x\left(t\right)-min\left(x_{group}\right)}{max\left(x_{group}\right)-min\left(x_{group}\right)}\right),$$

where

- *X_norm_* is the normalized value of x(t).
- $x_{accumulated}$ is the collected data from the beginning of the surgery to the current time.
- $x_{group}$ is the group dataset.

To compare the three methods, the residuals and correlation between the original parameters and their transformed values were compared. As the data were available offline, we assumed that a perfect normalization would be achieved by normalizing the data while minimizing both intra- and inter-patient variability. Therefore, the normalization of the offline data to obtain a perfect normalization was fixed with a weight of 0.7 for the individual data, as we already had the data, and with a weight of 0.3 for the group data. The obtained values were then compared to the online implementation of the normalization. The comparison was performed by calculating the MAE and correlation between the normalized data. The best method was selected based on a metric score; the correlation and MAE were normalized and given a percentage based on their values. As both MAE and correlation were equally important, the score was calculated as follows:

(4) $$Score(\%)=\frac{\left(Correlation_{normalized}+MAE_{normalized}\right)}{2}$$

### 2.4. Deep Learning Training

**Figure 7:**
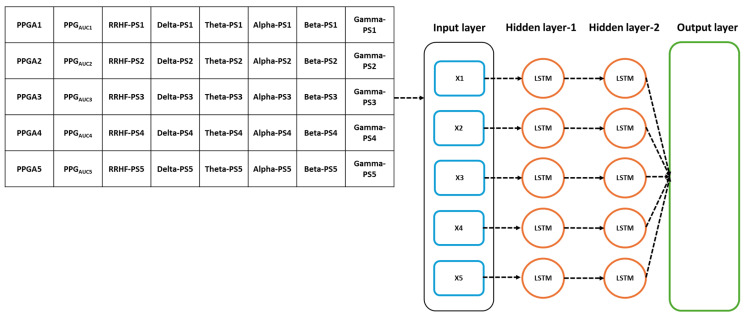
Details of the used LSTM model. The input parameters consist of 8 features, with a time step of 5 points. The data were originally processed in segments of 64 s with 59 s overlapping, which means that to predict one value, the model needs to wait first for 64 s to be processed before the prediction is made, and after the first window, it only waited for 5 s. PS refers to the power spectral estimation.

**Figure 8:**
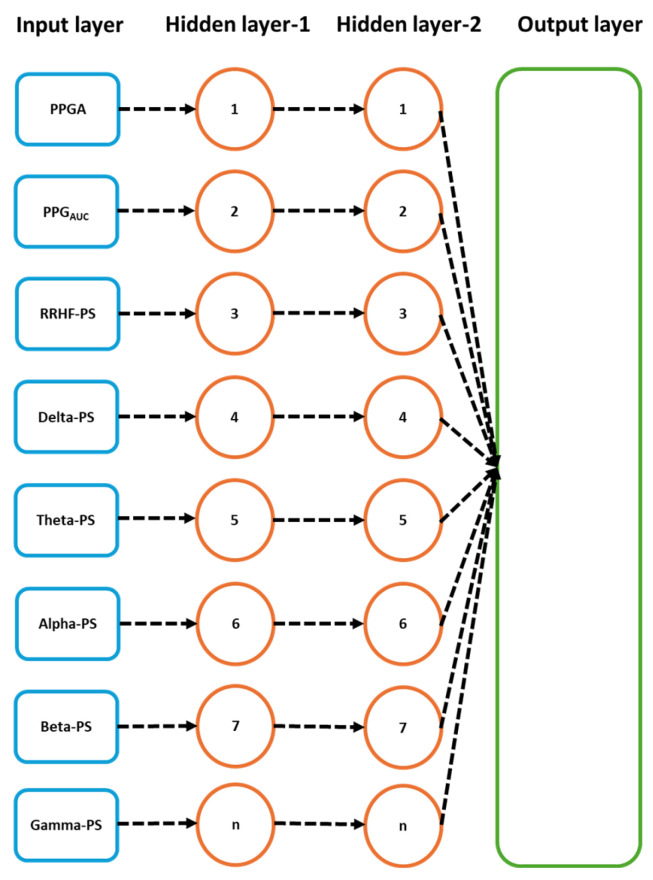
Details of the used neural network model. The input parameters here are one input per five seconds. After the first 64 s segment is available, it results in one prediction, and the segment is then updated by the next available 5 s data.

The models’ parameters were selected after applying an optimizable approach. The used models were MLP and LSTM. The models hyperparameters are listed in Table 2. The input parameters for the model were the five spectral power variables extracted from EEG signals, the spectral power of RRHF series, and the PPGA and PPG_AUC_ series. We first assumed that the model did not need to be complex; however, to cover every possibility, we set the number of hidden layers to be between 2 and 10. The learning rate was selected between 0.1 and 0.0001, and the batch size was random between 64 and 512. The selected optimal parameters are described in Table 2. Figure 7 and Figure 8 show the models’ architecture.

### 2.5. Statistical Analysis

Three tests were applied to measure the agreement between each doctor’s assessment. Analysis of variance (ANOVA) with a 0.05 significance level was used to test the similarity in the distributions and variance. To ensure consistency and agreement between the experts’ assessments, we applied the Bland–Altman test [26] and intraclass correlation (ICC) [27] with a 0.05 significance level. The ICC test was performed on individual assessments and on the whole data. The correlation coefficients and *t*-tests were calculated for pain assessments and the parameters used in this study. The used models were evaluated using the mean squared error (MSE) and R^2^ on the training and validation datasets. The test (10 patients) dataset was evaluated using the mean absolute error (MAE) and correlation analysis. For the ECKH data of 10 patients, we compared the differences of the mean at three different surgical stages: Intubation (t_1_), Incision (t_2_), and Extubation (t_3_). The receiver operating characteristic curve (ROC) was used to evaluate the models’ output in predicting nociception at the defined events for each patient individually (ECKH, *n* = 10).

## 3. Results

### 3.1. NOA

**Figure 9:**
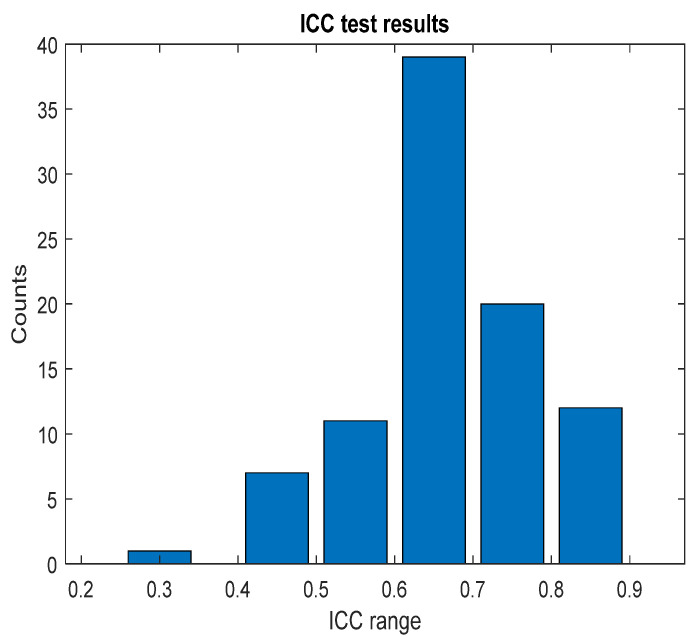
ICC test results. The figure shows the ICC results across each patient individually; the bar values (*y*-axis) represent the counts. The ICC result was in the corresponding range (*x*-axis).

**Figure 10:**
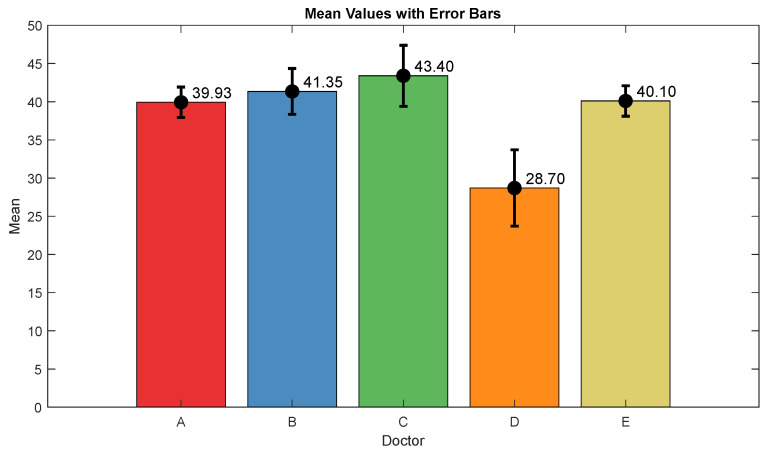
ANOVA test results. The mean value and interval plots for each doctor are shown in the figure.

**Figure 11:**
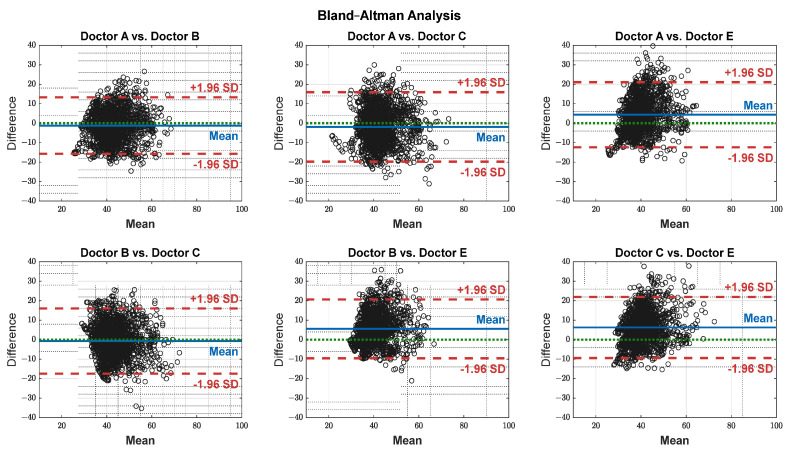
Agreement on a pairwise basis between each doctor’s assessment. The black dots represent the difference between each paired data points (*y*-axis) against their mean (*x*-axis). The blue horizontal lines represent the bias (mean difference), while the red dashed lines indicate the 95% limits of agreement (±1.96 SD). The green dotted lines represent zero bias (ideal).

The pain assessments from each doctor were processed carefully. As five anesthesiologists provided the assessments, we first used the ANOVA test. Doctor D provided the lowest assessment, and it was significantly different than the other four doctors [28]. Therefore, we only used assessments from Doctors A, B, C, and E. Moreover, we applied Bland–Altman and ICC [29] tests. When the pain assessments from all four doctors were pulled together, the ICC was 0.73 with a confidence interval of [0.69 0.75]. The ICC was also applied to each patient’s assessments individually. Figure 9 shows a histogram of the ICC values among the patients, and Figure 10 shows the ANOVA test results. The agreement between the doctors’ assessments was evaluated using Bland–Altman analysis and pairwise comparisons, with the results shown in Table 3 and Figure 11. The analysis results were consistent across the doctors in terms of agreement, where all data fell within the 95% limits of agreement (LoA). The lowest bias (−0.7) was observed between Doctors B and C, indicating minimal differences in their assessments. The highest difference was found between Doctors C and E (bias = 6.25) and Doctors B and E (bias = 5.55). The standard deviation of the differences ranged from 7.4 to 9.1.

### 3.2. Parameter Correlations

**Figure 12:**
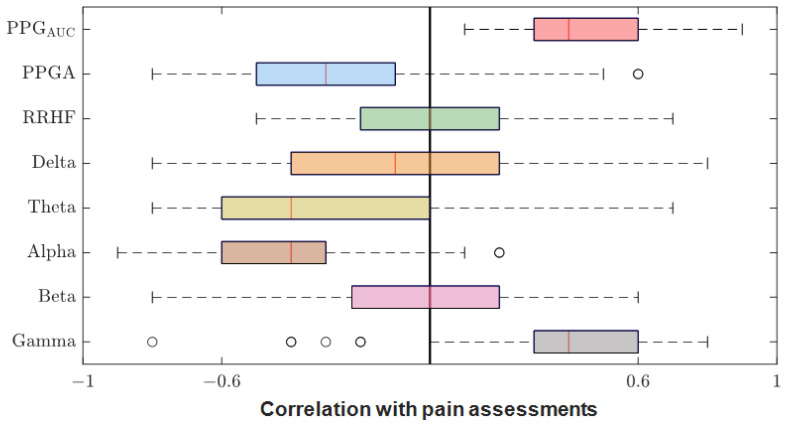
Correlation coefficients between parameters and pain assessments. The plot shows the distribution of the correlation coefficients for the 90 patients. The median is shown in the box (the red vertical line). The high and low quartiles are shown, and the outliers are marked with black open circles.

After we matched the NOA for the four doctors, the average was taken for further analysis. The Pearson correlation coefficient between the parameters and the average assessment were calculated for each patient individually. The results were pulled together and are shown in Figure 12. PPG_AUC_ had the highest individual positive correlation (0.85) and mean (0.46). The PPGA correlation had a mean value of −0.32; it was mostly negative, with the highest value of −0.82. The power calculations for EEG bands correlated negatively in most cases except for gamma, which had a close mean value (0.40) to PPG_AUC_. RRHF_PS_ had the lowest correlation along with the beta band (−0.003 and −0.004 mean values, respectively).

**Figure 13:**
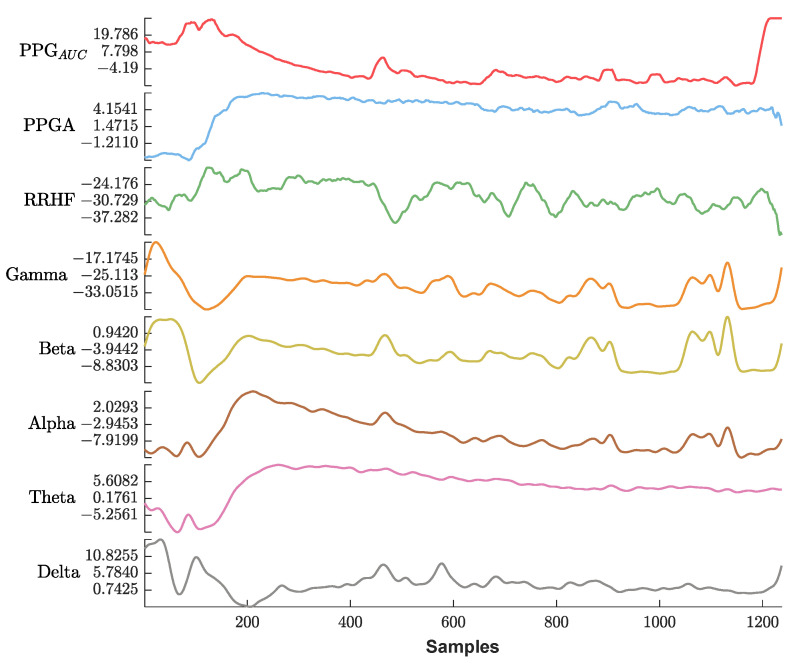
An example of the parameters used in this study. The *x*-axis is the sample index, and the *y*-axis is the parameter value. The EEG bands and RRHF are in (dB) power spectral. Waveforms are represented using different colors from top to bottom (PPG_AUC_, PPGA, RRHF, Gamma, Beta, Alpha, Theta, and Delta).

**Figure 14:**
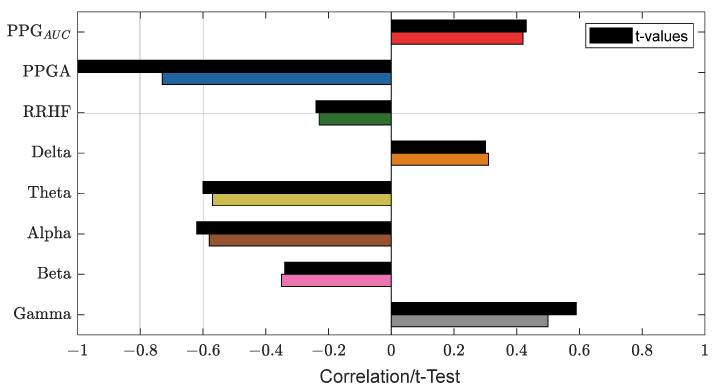
An example of the correlation analysis for one patient. The correlation coefficients are shown in different bar colors, and the *t*-test values with a significance level of 0.05 are in black bars. The plot shows PPGA with the highest correlation value (−0.73).

Figure 13 shows an example of the used parameters, where the curves show a good positive or negative correlation with the pain assessment. The correlation analysis for the same subject is shown in Figure 14.

### 3.3. Parameter Normalization

**Figure 15:**
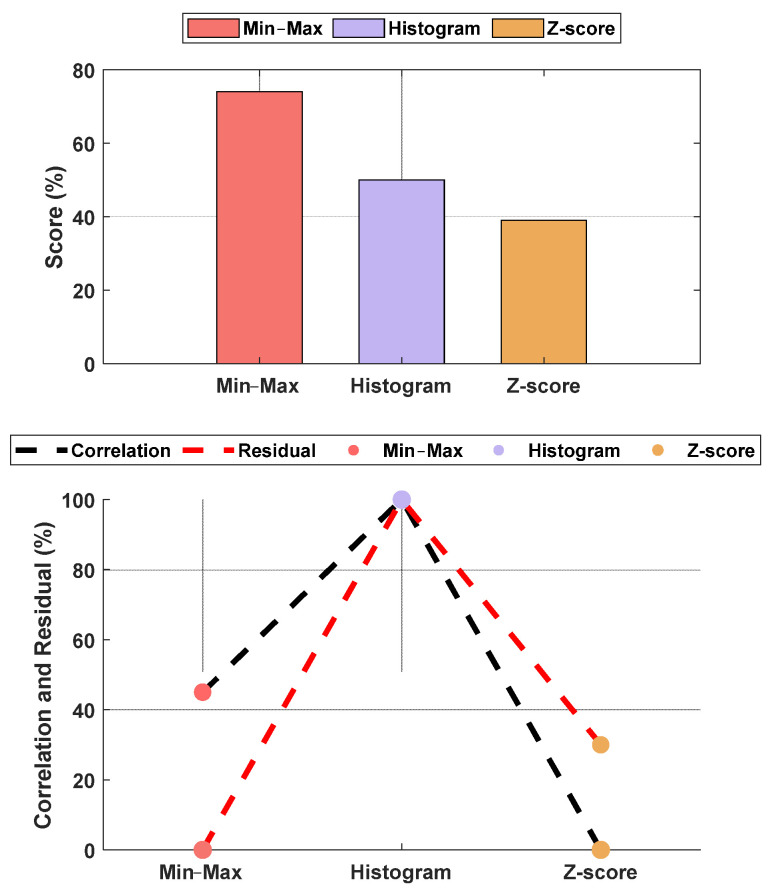
Performance comparison between normalization methods against the raw parameters. The **upper plot** is the score based on the normalized MAE and correlation, and the **lower plot** shows the normalized metrics. The figure shows Min–Max normalization with the lowest MAE and histogram normalization with the highest correlation. The overall score shows the Min–Max normalization with the best score, and, therefore, it was used in our algorithm. The raw parameter was normalized as if all data were already available. Therefore, the weights w_1_ and w_2_ were fixed at 0.7 for the individual (accumulated) dataset and 0.3 for the group dataset.

**Figure 16:**
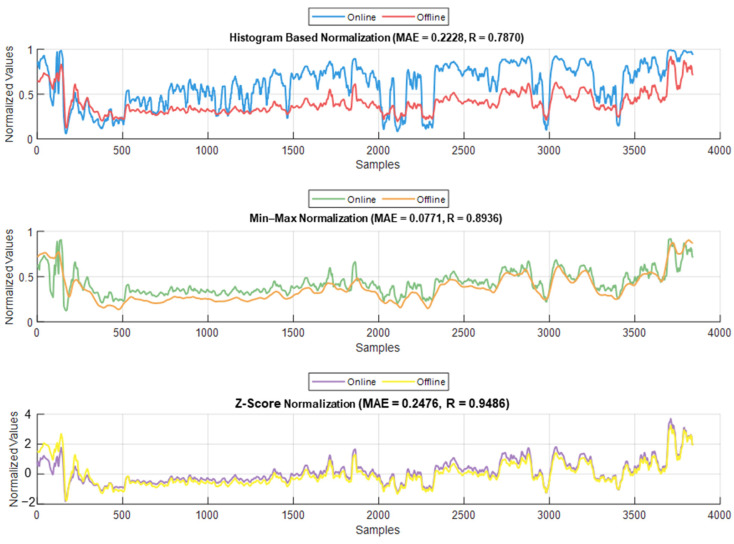
Example of the online and offline normalization for one patient (PPG_AUC_). The figure shows how histogram normalization either maximized or minimized the parameter value in online implementation, whereas Min–Max and z-score maintained results closer to the offline implementation.

The normalization methods resulted in different MAE and correlation values. The lowest MAE was found for Min–Max normalization, and the highest correlation was found for histogram normalization. The results are shown in Figure 15. An example of normalization is shown in Figure 16. For this case, the lowest MAE (0.08) was associated with Min–Max normalization, and the highest correlation was 0.95 for z-score normalization.

### 3.4. Pain/Nociception Models

Initially, we had 90 patients’ datasets. When more datasets from another hospital (ECKH-2023 datasets) became available, we decided to assess the model from a medical and engineering perspective. For NTUH (2015), the pain assessments were based on anesthetic records. However, these records were not easily available to us at the moment of this study; therefore, we were unable to assess the 10 patient test datasets from a medical perspective. Thus, the NTUH data were evaluated using MSE and R^2^ on the training and validation datasets and correlation analysis and MAE on the test dataset. For ECKH, the needed information, such as painful stimuli events, was available; therefore, medical analysis was possible.

#### 3.4.1. MLP Model

**Figure 17:**
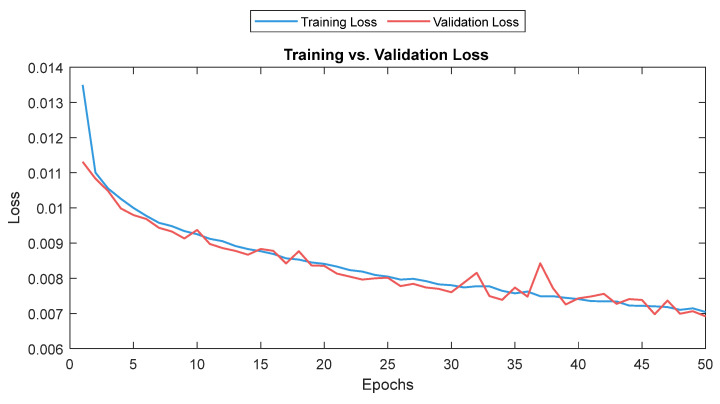
Training and validation loss for the MLP model. The training and validation loss curves are shown as a solid and a dashed line, respectively.

**Figure 18:**
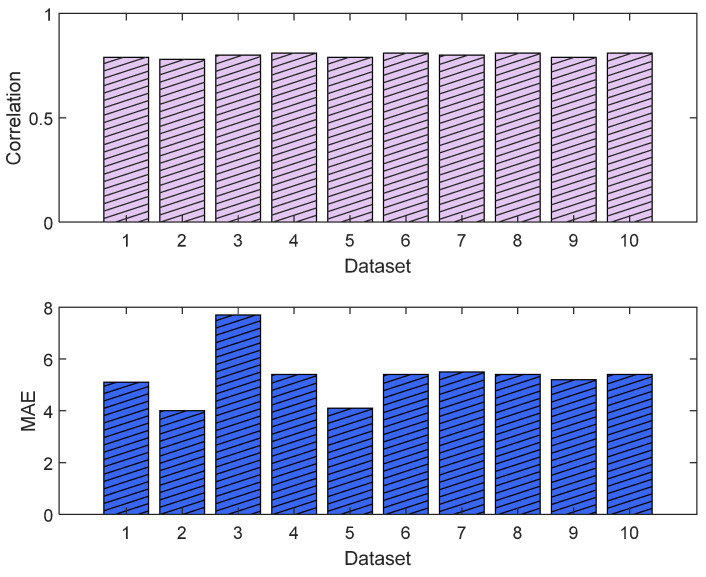
Correlation analysis and error results (MLP). The **upper plot** shows the distribution of the correlation values across the test dataset (NTUH) with the pain assessments, and the **lower plot** shows the MAE results.

The MLP losses were 0.008 and 0.007 on training and validation, respectively. The R^2^ was 0.82 on training and 0.80 on validation. For the test dataset (NTUH), the mean values for correlation and MAE were 0.78 and 5.1, respectively. Figure 17 shows the curves for training and validation, and Figure 18 shows the distribution of the correlation and MAE values for the test datasets.

**Figure 19:**
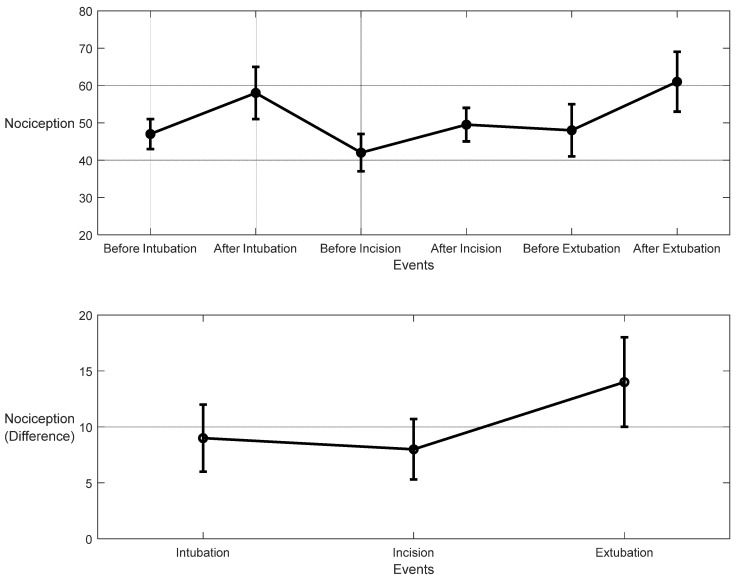
Nociception reactions at different events during surgery. These values were obtained by the MLP model. The **upper plot** shows the distributions before and after the event for the 10 patients together. The *x*-axis is the time at which a surgical stimulus happened, and the *y*-axis is the distribution of the values during the time before and after (the whiskers) the event. The mean value for each event is shown by the closed circle. The **lower plot** shows the difference in means before and after the events. The error bars represent the standard error, and the closed circles are for the difference in means. The *p*-value for all events was *p* < 0.01 using the Mann–Whitney test. The difference in means (SEs) were 9 (3), 8 (2.7), and 14 (4).

For the 10 patients’ datasets from ECKH, the mean value before and after the three defined events was calculated. The mean values after t_1_ increased from 48 to 57. While at t_2_, the mean value increased the least, from 41 to 50, and at t_3_, the mean value increased the most, from 48 to 62. Figure 19 shows the changes in the mean values before and after each event (upper plot) and the difference in the mean NOA at each event (lower plot). The upper plot shows how the mean values before and after each event varied across the 10 patients. The lower plot demonstrates the differences in the mean NOA at each event between the patients. The highest difference was found at t_3_, with a value of 13, and the lowest was at t_2_, with a value of 8. The difference in the NOA means at t_1_ was also clearly noticed, with a value of 9.

**Figure 20:**
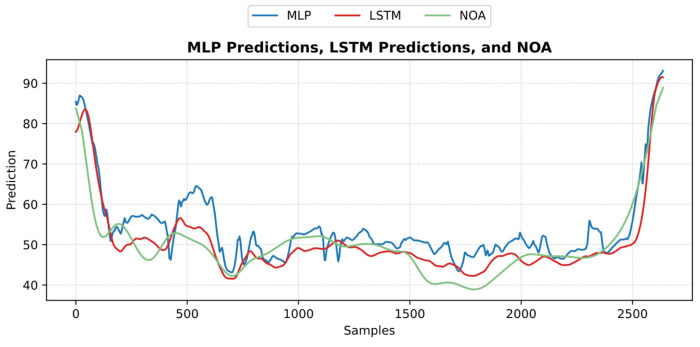
An example of the predicted nociception from the NTUH test dataset. The figure shows how both predictions implied a strong positive correlation and low error with pain assessment.

An example of the predicted pain/nociception from the two models is shown in Figure 20 (NTUH dataset).

#### 3.4.2. LSTM Model

**Figure 21:**
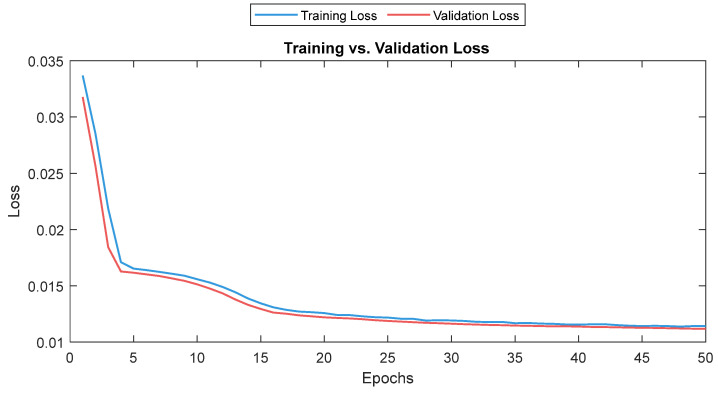
Training and validation loss for the LSTM model. The training and validation loss curves are shown as a solid and a dashed line, respectively.

**Figure 22:**
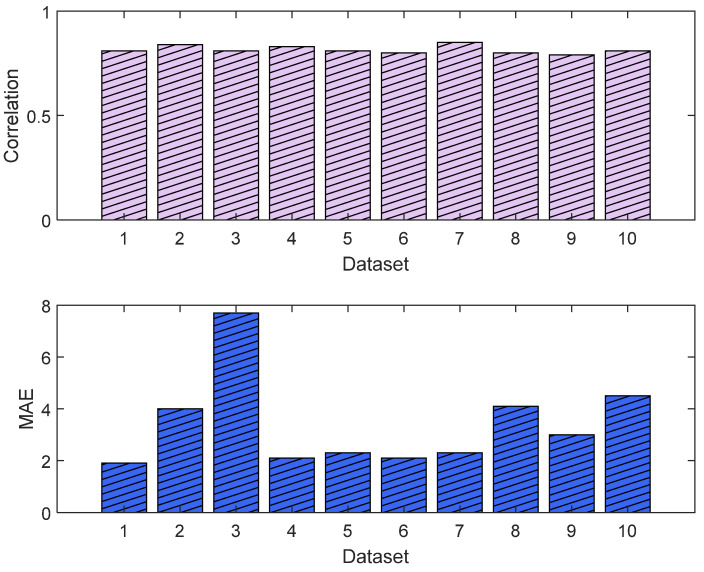
Correlation analysis and error results. The **upper plot** shows the distribution of the correlation values across the test dataset (NTUH) with the pain assessments, and the **lower plot** shows the MAE results.

The training and validation losses for the LSTM model were 0.012 and 0.011, respectively. The R^2^ was 0.87 and 0.81 on the training and validation, respectively. The mean value of the correlation and MAE were 0.85 and 3.6, respectively. Figure 21 shows the trends of the training and validation. Figure 22 shows the correlation and MAE values for each patient. The results were consistent, except for patient 3, who had a high MAE of 7.8.

**Figure 23:**
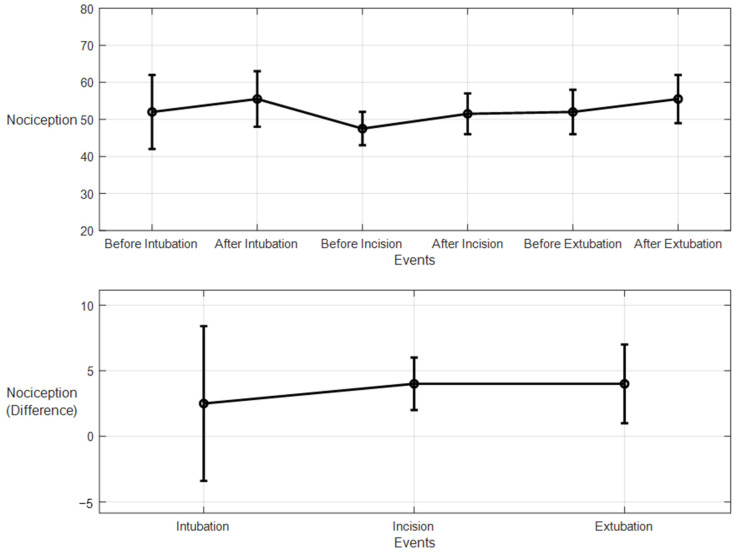
Nociception reactions at different events during surgery (LSTM model). The **upper plot** shows the distributions of the mean values before and after the event for the ten patients together. The *x*-axis is the time at which a surgical stimulus happened, and the *y*-axis is the distribution of the values during the time before and after (the whiskers) the event. The mean value for each event is shown by the closed circle. The **lower plot** shows the difference in the means before and after the events. The error bars represent the standard error, and the closed circles represent the difference in means. The *p*-value for all events was *p* < 0.01 using the Mann–Whitney test. The differences in the means (SEs) were 2.5 (5.9), 4 (2), and 4 (3).

For the 10 patients’ datasets from ECKH, the mean values before and after the three defined events increased less than the MLP model. The mean values before and after t_1_ were 53 and 55, respectively, and it was the lowest. The mean values before and after t_2_ were 47 and 51, respectively, and at t_3_, the values were 51 and 55 before and after, respectively. Figure 23 shows the changes in the mean values before and after each event (upper plot) and the difference in the mean NOA at each event (lower plot). The upper plot shows how the mean values before and after each event varied across the 10 patients. The lower plot demonstrates the differences in the mean NOA at each event between the patients. The highest difference was found at t_2_ and t_3_, with a value of 4, and the lowest was at t_1_, with a value of 2.5.

**Figure 24:**
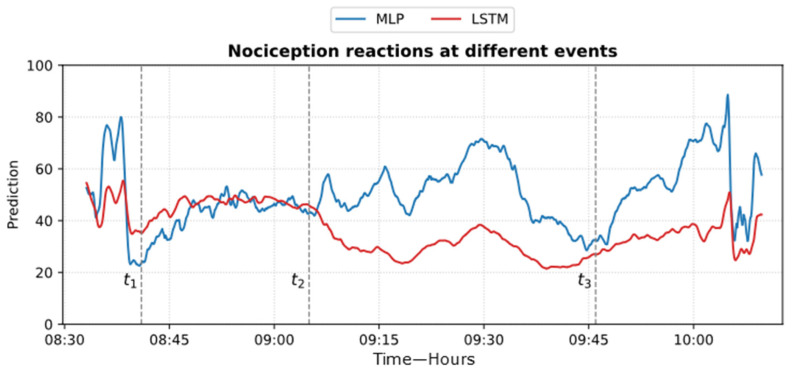
Example of MLP and LSTM predictions from ECKH. The 3 defined events are marked with vertical dashed lines. The figure shows how the MLP predictions reacted better at all events than LSTM.

Figure 24 shows an example of the models’ predictions on the ECKH dataset.

**Figure 25:**
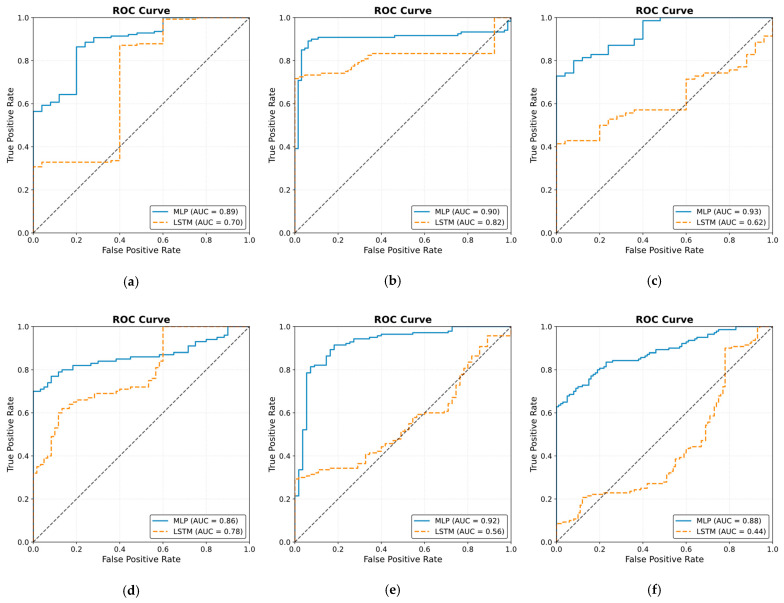

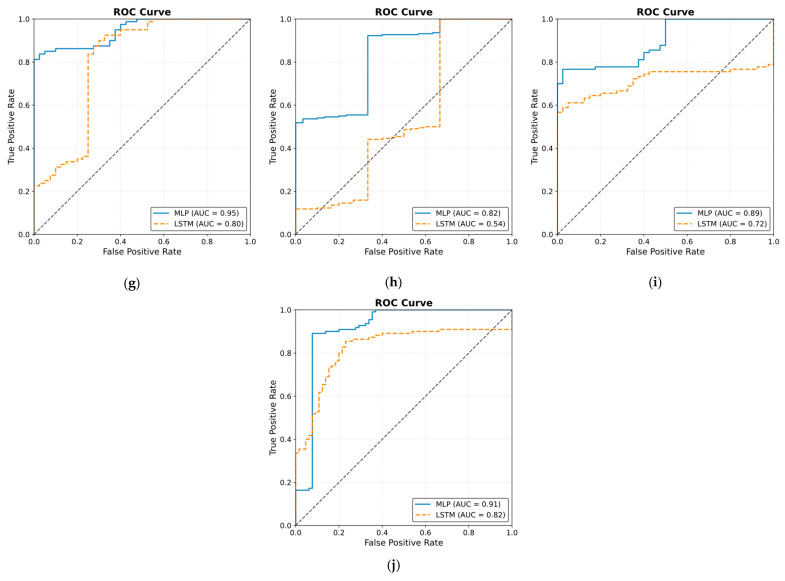
Receiver operating curve (ROC) results. The ROC illustrates the ability of the two models to distinguish noxious stimuli at t_1_, t_2_, and t_3_. The subfigures from (**a**–**j**) correspond to individual patients, ordered sequentially, where each subfigure presents the ROC curve for the respective patient. The black dashed line in each ROC plot represents the performance of a non-discriminatory or random classifier. The results show MLP with more reactivity and ability to differentiate noxious stimuli, where the mean value for the area under the curve (AUC) across the 10 patients was 0.90.

The ROC curve results are shown in Figure 25. The results were obtained by classifying NOAs based on the events. The figure shows that the MLP model outperformed the LSTM model in predicting noxious stimuli. The mean value for MLP AUC was 0.90; the highest was 0.95, and the lowest was 0.82. The LSTM demonstrated low to moderate reactivity; the mean AUC value was 0.68, whereas the lowest and highest values were 0.44 and 0.82, respectively.

## 4. Discussion

Many indexes were developed specifically to monitor the nociception–antinociception balance during general anesthesia [3,5,6,10,11,12,30], and all were based on different parameters or a combination of those parameters (e.g., PPGA, HBI, EEG features). All of them have proven utility and were able to react to painful stimuli events. Single-source indexes were reported to react to other factors that do not relate to nociception [4]. On the other hand, we believe that single-source indexes might ignore other information that could be helpful to assess NAN. Therefore, in our study, we developed two models to predict nociception in various stages of surgery. First, the ANOVA test was used to check the distributions and variance in the assessments among the doctors. Doctor D showed disagreement with the others; therefore, the related assessments were excluded. To further prove that the other doctors’ assessments were consistent and reliable, we applied ICC and Bland–Altman tests on the assessments. The ICC was applied to the data when pulled together and individually. In both cases, the results proved the reliability between the assessment, as shown in Figure 9 (0.73 on the whole data). The Bland–Altman test also showed that 95% of the data was in the upper and lower limits of agreement (Table 3 and Figure 11); therefore, the assessments were used in further analysis. The parameter selection was based on the clinical parameters that were previously used to assess NAN [3,5,10,11]. PPGA and HBI were used for SPI development in a single-source model [3] and in a multimodal approach [11], while EEG parameters were used in other methods [7,8,10,31]. A correlation analysis was applied to the selected parameters with the pain assessments, and the results proved the validity of our pain assessments. The SPI was calculated using the equation (SPI = 100 − (0.7 × PPGA + 0.3 × HBI), where PPGA has the most contribution. This equation implies a negative correlation between nociception and PPGA or HBI, which was the case in our analysis, where PPGA correlated negatively with the assessments and the values were distributed in the range of −(0.2–0.82), as shown in Figure 12. This agrees with the correlation analysis performed in [3] with pain stimulus intensity. The EEG parameters also showed an agreement with the results in [32,33], where the alpha band was reported to drop in the presence of painful stimuli. Other studies showed that the delta [34] and beta bands [35] increased with painful stimuli, and in our case, this was noted in many cases.

Inter- and intra-patient variability needs to be addressed carefully. Previously, methods such as histogram normalization and z-score normalization were adapted for online usage. In our study, both methods were effective; however, we noticed that some parameters’ properties were affected after applying those techniques. Therefore, we adapted another normalization method (i.e., Min–Max normalization) for our use. The comparison between the three methods showed that for our data, the Min–Max method is the most effective in retaining the original parameter properties while reducing both inter- and intra-patient variability.

In our study, both models used information from many sources, and both were based on experts’ assessments of pain. The models were evaluated in two groups: the first group was used to evaluate the models’ performances with the pain assessments (NTUH), and the second group was used to evaluate the models’ performances in the operation room (ECKH). On the NTUH datasets, the MLP model had a higher MAE and lower correlation compared to the LSTM model (Figure 18 and Figure 22). When the predictions were visualized, however, we noticed that both models had predictions that generalized well around the pain assessments, with values from the LSTM model being lower and smoother. This difference was clear, and the LSTM predictions were not conclusive whether the current predicted value was related to nociception or not. On the other hand, the MLP predictions were more precise about the current state, whether it was related to nociception or not. This was proven in the prediction from the ECKH datasets. The predicted values before and after surgical events were compared. For the MLP model (Figure 19), the mean value after t_1_ and t_3_ increased the most, and it was more spread out after t3 (52–69). The mean value of the difference before and after each event provided evidence that the MLP model was able to predict nociception at painful stimuli events, where it was 9 and 12 at t_1_ and t_3_, respectively. While smaller than t_1_ and t_3_, the increase after t_2_ was still noticed; this was also proven in the difference before and after t_2_, where the mean value was 8. On the other hand, for the LSTM model (Figure 23), the mean values of the predicted nociception before and after each event were not large enough to distinguish between different levels of nociception. The largest increase in the predicted value was noticed after t2 and t3, where the difference between the means before and after the two events was also small (4).

The obtained results in Figure 19 and Figure 23 provide insights into the models’ behaviors at the defined events. In particular, the difference in the NOA mean at each event describes the ability of the model to distinguish a noxious stimulus. This was further proven by applying ROC on the defined events (Figure 25). The MLP model demonstrated more accurate predictions, achieving a mean AUC of 0.90 (range 0.82–0.95). Hence, this indicates a distinctive assessment of nociception at various stages of surgery. In contrast, the LSTM model provided more variable performance, with a mean AUC value of 0.68 (range 0.44–0.82). Considering the metrics used in our study, there is a clear gap in the performance of the two models that was noticed when evaluating the ECKH dataset. This could be related to different factors, including the complexity of LSTM models. Our iterative approach in training the LSTM model did not improve the results. The selection for LSTM was based on the fact that it considers previous knowledge, which could be beneficial in some cases. In our case, however, this might have been one of the limitations to our model. Moreover, we hypothesize that the significant difference in the models’ performances could be related to the fact that MLP models could capture hidden patterns and complex relationships accurately.

**Figure 26:**
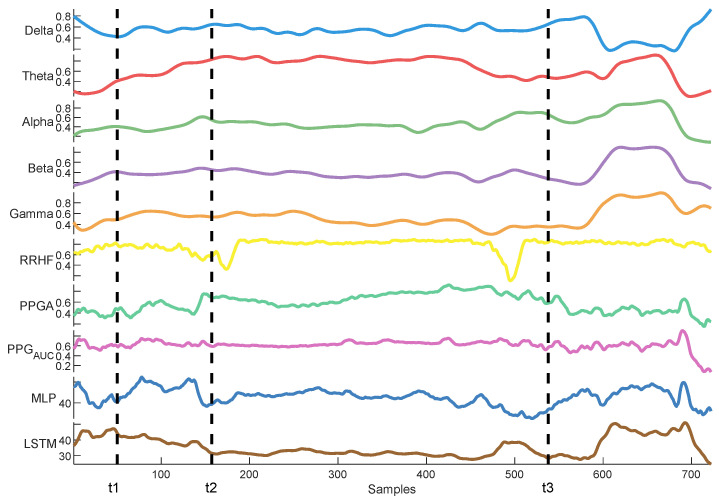
An example of parameter trends against LSTM and MLP predictions. The figure illustrates how each model behaved differently according to the parameters’ changes, with the MLP model demonstrating better reactivity.

Delta arousal (power increase) and alpha dropout (power decrease) are related to noxious stimuli. In Figure 26, we show how every single parameter reacted during surgery against MLP and LSTM predictions. At t_1_, MLP predictions increased, which corresponded to delta arousal and alpha dropout. At t_2_, less reactive behavior was noticed from alpha and delta; similarly, PPGA smoothly decreased, and changes were not noticed in PPG_AUC_. Although MLP predictions reacted to these features, resulting in higher predictions, the LSTM model was unable to react to these changes. At t_3_, small changes in delta and alpha and a slow decrease in PPGA were noticed. Unlike the LSTM model, the MLP model was able to react to these changes. These results highlight the need for multiple parameter approaches. A model utilizing one parameter might ignore other information. An increase in PPGA is related to analgesia. As seen in the figure between t_1_ and t_2_, PPGA increased in this time segment; this increase could be related to other factors. In the absence of other parameters, a model would predict this value as a decrease in nociception; however, in our case, the usage of multiple parameters provided sufficient data to the MLP model, which identified this as a noxious stimulus.

Our study aimed to enhance nociception monitoring in general anesthesia by developing a multimodal framework that integrates multiple physiological parameters, offering more comprehensive information for nociception assessment. We applied a comparative analysis of two models (MLP and LSTM) for nociception prediction, demonstrating their relevant strengths and limitations in different clinical settings. Our evaluation across two hospitals further supports the generalizability of these findings and the ability of our MLP model to be used in different clinical settings and patient populations. Our analysis for the used signals demonstrated the capability of EEG signals as an important source for pain assessment, which was further validated with the models’ results. Additionally, utilizing Min–Max normalization for online and offline usage provided better results than histogram and z-score normalization. This was found when the correlation analysis was applied to the pain assessments with the used parameters. Initially, the raw parameters correlated to pain assessments; however, histogram normalization affected these results negatively. Exploring other methods used in the literature, such as the z-score, led to using another common normalization method (i.e., Min–Max normalization).

Our analysis for model development focused on traditional regression metrics (MSE, MAE, and R^2^), given the continuous nature of our datasets. For model validations, a statistical comparison in the mean difference between events was utilized, and the models’ abilities to differentiate nociceptive events were tested using ROC curves. However, we acknowledge the importance of including performance metrics such as F1, precision, and recall. These metrics could be adopted in the future with larger datasets and various anesthesia settings.

Our previous studies about pain/nociception did not achieve this performance. We used SPI [29] and ANI [28] features to predict pain scores using LSTM and MLP models. The best model [29] had a correlation of 0.63 and an MAE of 3.42; however, despite this difference in performance, the predictions did not generalize well to the pain assessments, which was due to insufficient information provided to the model. Hence, we solved this in our present study, and the results improved.

The types of surgeries and anesthesia administration methods could have an effect on the way the model predicts nociception. Our datasets were a combination of inhaled anesthesia and, in a few cases, TCI anesthesia. In both cases, propofol was used intravenously. Therefore, the findings in this study are more related to inhaled anesthesia. The MLP model was more useful in distinguishing the nociceptive reactions at different surgical events. However, this was noticed only when the models were evaluated on ECKH datasets. Considering the size of the dataset (10 patients), the size of the test dataset might not be enough to prove this. Therefore, future studies are needed to validate this result.

## 5. Conclusions

We developed two multimodal models for nociception prediction in general anesthesia. The models use physiological parameters from various sources rather than relying solely on one signal’s information. The developed models were correlated with an expert assessment of pain that was based on clinical information related to nociception and surgical stimulation events. Our investigation of normalization techniques revealed that Min–Max normalization was optimal for both online and offline applications, preserving essential parameter characteristics. The models were evaluated across two hospitals with varying results. In the first group, both models demonstrated effective nociception prediction, with the LSTM model providing smoother predictions. In the second group, while LSTM models are typically powerful for sequence data problems, this advantage did not translate to better performance in our specific application. The MLP model successfully differentiated between nociception levels at various events, while the LSTM model’s predictions were less definitive. Our results are promising; however, further studies are needed to validate these findings in different anesthesia settings and a larger patient population.

## Figures and Tables

**Table 1:** Patient demographic. Fentanyl and propofol were administered intravenously.

	NTUH		ECKH	
	Range	Mean ± SD	Range	Mean ± SD
Age (yr)	22–78	48 ± 12	40–67	57 ± 10
Weight (kg)	40–160	59 ± 14	41–93	68 ± 18
Height (cm)	138–185	157 ± 7	150–189	166 ± 15
Fentanyl IV (µg)	50–205	117 ± 42	50–100	80 ± 27
Propofol IV (mg)	50–250	124 ± 30	90–200	122 ± 45

**Table 2:** Models’ specifications. The activation within the hidden layers were, by default, sigmoid for the LSTM model and ReLU for MLP.

	Hidden Layers	Neurons	Batch Size	Epochs	Learning Rate	Optimizer	Activation (Output)
MLP	2	50/30	125	50	0.001	Adam	ReLU
LSTM	2	100/200	256	50	0.0001	Adam	ReLU

**Table 3:** Bland–Altman test results.

Group1	Group2	SD	Bias	Agreement (%)
A	B	7.40	−1.22	95.00
A	C	9.11	−1.92	96.00
A	E	8.52	4.32	95.00
B	C	8.55	−0.7	95.00
B	E	7.70	5.55	95.01
C	E	8.01	6.25	95.00

## Data Availability

Data presented in the paper are available on request from the corresponding authors S.-Z.F. and J.-S.S.
